# ConFERMing the role of talin in integrin activation and mechanosignaling

**DOI:** 10.1242/jcs.260576

**Published:** 2023-04-20

**Authors:** Michael Bachmann, Baihao Su, Rolle Rahikainen, Vesa P. Hytönen, Jinhua Wu, Bernhard Wehrle-Haller

**Affiliations:** ^1^ Department of Cell Physiology and Metabolism, Centre Médical Universitaire, 1211 Geneva 4, Switzerland; ^2^ Molecular Therapeutics Program, Fox Chase Cancer Center, 333 Cottman Ave, Philadelphia, PA 19111, USA; ^3^ Faculty of Medicine and Health Technology, Arvo Ylpön katu 34, Tampere University, FI-33520 Tampere, Finland; ^4^ Fimlab Laboratories, Biokatu 4, FI-33520 Tampere, Finland

**Keywords:** Adhesion, Signaling, Kindlin, Rap1, Paxillin, RIAM, PIP2, Vinculin, Actin

## Abstract

Talin (herein referring to the talin-1 form), is a cytoskeletal adapter protein that binds integrin receptors and F-actin, and is a key factor in the formation and regulation of integrin-dependent cell–matrix adhesions. Talin forms the mechanical link between the cytoplasmic domain of integrins and the actin cytoskeleton. Through this linkage, talin is at the origin of mechanosignaling occurring at the plasma membrane–cytoskeleton interface. Despite its central position, talin is not able to fulfill its tasks alone, but requires help from kindlin and paxillin to detect and transform the mechanical tension along the integrin–talin–F-actin axis into intracellular signaling. The talin head forms a classical FERM domain, which is required to bind and regulate the conformation of the integrin receptor, as well as to induce intracellular force sensing. The FERM domain allows the strategic positioning of protein–protein and protein–lipid interfaces, including the membrane-binding and integrin affinity-regulating F1 loop, as well as the interaction with lipid-anchored Rap1 (Rap1a and Rap1b in mammals) GTPase. Here, we summarize the structural and regulatory features of talin and explain how it regulates cell adhesion and force transmission, as well as intracellular signaling at integrin-containing cell–matrix attachment sites.

## Teaser

**Summary:** Talin is a key protein controlling integrin-dependent cell–matrix adhesions. Here, we summarize its function in linking mechanical coupling to the actin cytoskeleton and adhesion signaling.

## Introduction

Talin (herein referring to talin-1 unless otherwise noted; see below), was first reported as P235, and identified in human platelets as a dimeric, globular and highly abundant protein (3–8%) with a molecular mass of 235 kDa (Collier and Wang, 1982). Purified P235/talin bound and cross-linked filamentous actin and was identified in parallel by Burridge and Connell in fibroblasts; they named it talin (Burridge and Connell, 1983). Talin localized to the ends of stress fibers in focal adhesions and showed the ability to bind vinculin (Geiger et al., 1984; O'Halloran et al., 1985). Subsequently, talin was observed to bind to detergent-extracted integrin complexes (Horwitz et al., 1986), and to link membranes to actin filaments (Isenberg and Goldmann, 1992; Niggli et al., 1994). A few years later, talin was found to be recruited to ligand-bound integrins, linking ligand-induced conformational changes of integrins to talin binding in an ‘outside-in’ manner (Miyamoto et al., 1995). In leukocytes, ‘inside-out’ activation of integrins is induced in a Rap1-GTPase-dependent manner, whereas high concentrations of extracellular integrin ligands mediate outside-in activation independent of Rap1 (Rap1a and Rap1b forms in mammals) (Liu et al., 2002).

Another means by which talin regulates integrins was found to be through their α- and β-subunit association. Integrin mutations at the juxtamembrane region led to spontaneous activation of the αIIbβ3 integrin (platelet receptor) (Hughes et al., 1996). This proposed that resting integrins exhibited closely associated α- and β-subunits, negatively controlling access of intracellular adapters such as talin.

Therefore, new biochemical assays were developed to study the direct interaction between β-integrin tails and different cytoplasmic adapters (Pfaff et al., 1998).

Overall, it became clear that integrins are regulated by an interplay of competition and interaction between many cytoplasmic adapters; among these, talin stood out because of its integrin-activating function (Calderwood et al., 2001; van der Flier et al., 2002; Soto-Ribeiro et al., 2019; Kiema et al., 2006; Takala et al., 2008).

With the recording of conformational changes in the αIIbβ3 integrin, critical residues were identified in the integrin and in the talin N-terminal domain that controlled talin-mediated integrin activation (Calderwood et al., 1999; Tadokoro et al., 2003). In parallel, Mn^2+^-induced integrin clustering in cells plated on immobilized integrin ligands allowed the definition of a role for acidic phospholipids, such as phosphatidylinositol(4,5)P_2_ (PIP2) in talin-mediated integrin activation, clustering and cell adhesion (Cluzel et al., 2005). Similarly, Mn^2+^-induced integrin-clustering was increased by the talin head and occurred independently of polymerized F-actin and other focal adhesion components, such as vinculin, paxillin and FAK (also known as PTK2) (Bachmann et al., 2020; Cluzel et al., 2005). With the development of talin-depleted cells and animals, the role of talin was identified to be two-fold: first, the talin head induced integrin activation and isotropic cell spreading, and second, the talin C-terminal (rod) domain formed the mechanical link to actin stress fibers, leading to the formation of focal adhesions, assuring essential functions during development and platelet function (Monkley et al., 2000; Nieswandt et al., 2007; Petrich et al., 2007; Zhang et al., 2008). On the one hand, this demonstrated that the rod domain of talin interacted with F-actin and recruited vinculin in a tension-dependent manner (Atherton et al., 2015; del Rio et al., 2009; Franco et al., 2006; Gingras et al., 2008; Hytonen and Vogel, 2008; Rahikainen et al., 2017; Senetar et al., 2004; Smith and McCann, 2007). On the other hand, the N-terminal head of talin was shown to regulate integrin conformation and extracellular matrix (ECM) binding (Cluzel et al., 2005). However, both parts of talin, and tensional forces were required to recruit signaling adapters such as paxillin to the integrin–talin complex (Sawada and Sheetz, 2002; Vogel and Sheetz, 2009).

However, the structure of the talin head domain remained controversial. Although the talin head has homology to FERM-domain-containing proteins, it was the only structure of such FERM domains that did not fold into the canonical compact ‘FERM cloverleaf’ structure (see below). Recently, we presented the first talin head structure that demonstrated the ability of the talin head to fold into this FERM cloverleaf, together with a possible explanation for why earlier structures folded into an extended, linear structure instead. In the remaining part of the review, we will name the FERM-folded talin head ‘compact’ (PDB 6VGU) (Zhang et al., 2020), in contrast to an atypical FERM structure, showing an ‘extended’ or ‘linear’ talin head (PDB 3IVF) (Elliott et al., 2010). We will discuss the consequences of the compact FERM fold of the talin head for integrin- and adapter binding, as well as regulation of integrin activity and F-actin association by talin.

## Similarities and differences of FERM-domain containing proteins in membrane and ligand binding

**Fig. 1.**
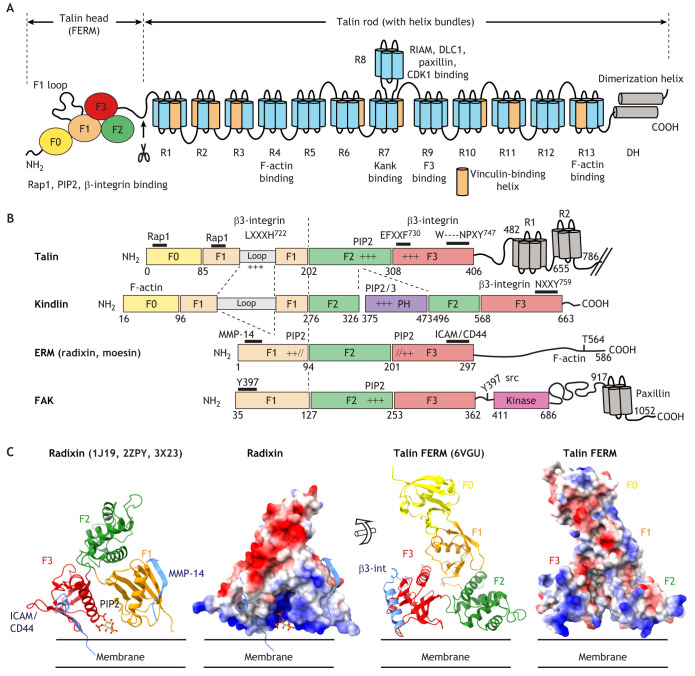
**Domain organization of talin and comparison to other FERM domain proteins.** (A) Domain structure of talin. A four-lobed head domain is joined to a cleavable linker to the C-terminal rod domain consisting of 13 helical bundles, each with either four or five helices, followed by the dimerization helix inducing antiparallel association. (B) Comparison between FERM proteins. Alignment of the talin FERM domain with that of kindlin, radixin and FAK. The different FERM-subdomains (with indicated positions) and insertions are color coded and aligned to the F1 and F2 subdomain junction. Identified peptide binding sites are indicated above the structural elements and highlighted with black bars. Basic binding sites for phosphoinositides are indicated with ‘+’ signs. The ‘//’ sign indicates that the positively charged patch contains a segment from F1 and another segment from F3, both contributing towards PIP2 binding. (C) 3D structure of the radixin and talin FERM domain. Ribbon model colored according to subdomains (left) and protein surface colored according to surface charge are shown (red is negative, and blue is positive charge). The binding pocket for PIP2, as well as ICAM-2 and CD44 ligands (binding to F3, PDB 1J19 and 2ZPY) (Hamada et al., 2003) and MMP-14 (binding to F1, PDB 3X23) (Terawaki et al., 2015) are indicated for radixin. Binding of β3 integrin (β3-int) is indicated for talin (PDB 6VGU). ChimeraX session files for all figures showing protein structures are available under https://doi.org/10.26037/yareta:eyekudd2rvab3oofli5auhoz7u, in order to facilitate the visualization and analysis of the 3D structures. Please note that radixin activation occurs by phosphorylation of T564 located in the C-terminal domain (B).

Talin exists as two highly similar isoforms (talin-1 and talin-2), with differences in expression pattern and functions in specific adhesion types that are not yet fully elucidated (Anthis et al., 2010, 2009; Monkley et al., 2001; Praekelt et al., 2012; Senetar et al., 2007). In this Review, we will use the term talin to describe the function of talin-1 if not otherwise stated. The N-terminal domain of talin has strong similarity to FERM-domain-containing proteins, whereas the C-terminal part (rod) contains multiple helical bundles that associate with F-actin and a variety of other binding partners (Fig. 1A). A FERM fold is characterized by a cloverleaf like arrangement of three subdomains called F1 (ubiquitin-like fold), F2 and F3 [the latter of which is similar to a phosphotyrosine-binding (PTB) domain]. In talin, as well as in kindlin (Li et al., 2017), the F1 subdomain is duplicated, forming a F0–F1 tandem (Goult et al., 2010; Li et al., 2017), which, according to our recent structure (Zhang et al., 2020), associates with the F2 and F3 subdomains to generate a compact structural arrangement that is very similar to the compact FERM fold shown in all available structures of other FERM-domain-containing proteins. Both talin and kindlin have an inserted loop in their F1 subdomain. Although the exact structure of this F1 loop in the context of the complete FERM domain has not yet been resolved, experiments in cells show that it is a site of talin-mediated integrin regulation (Goult et al., 2010; Kukkurainen et al., 2020) (see below).

Thus, talin is a bona fide FERM domain protein sharing multiple features with other FERM family proteins, including binding sites for lipids, peptides and F-actin. The latter function is shared with ezrin, radixin and moesin, which exhibit a C-terminal F-actin-binding domain that can be activated from the autoinhibited FERM-bound configuration by a phosphorylation switch (Fig. 1B,C).

FERM-domain-containing proteins often bind to juxtamembrane domains of single-pass cell surface receptors, such as CD44, ICAM-2 (Hamada et al., 2003; Mori et al., 2008) or β-integrins in the case of talin. This interaction is mediated via a binding site located in the PTB-related F3 subdomain. Similar to classical PTB domains, binding to FERM F3 occurs via a short anti-parallel β-strand followed by a turn that is often induced by a characteristic NPXY motif (Fig. 1B,C).

**Fig. 2.**
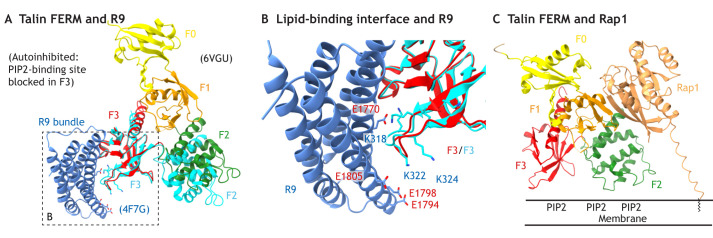
**Talin autoinhibition and activation via interaction with Rap1.** (A) Ribbon representation of the autoinhibited talin FERM–R9-bundle complex. The R9–F3 duplex (PDB 4F7G; R9, blue; F2-F3, cyan), is aligned onto F3 (red) in the FERM-folded talin head (PDB 6VGU; F0, yellow; F1, orange; F2, green; F3, red). (B) Enlarged view of the talin FERM–R9 complex pinpointing the PIP2-membrane-interacting basic loop in F3, and its shielding by R9. Labeled amino acids (red, acidic; blue, basic) are involved in R9–F3 interaction, and or F3–PIP2 interaction. (C) Ribbon model of the Rap1-bound talin FERM domain, showing Rap1 interacting with the F1 subdomain.

Lipid-binding orients FERM domain proteins on the plasma membrane (Fig. 1C). These binding sites are juxtaposed to the F3 subdomain, thereby optimizing its binding to transmembrane receptors (Fig. 1C). The exposure of lipid-binding sites in FERM domain proteins is a highly regulated process, whereby intramolecular autoinhibitory interactions prevent the constitutive association of FERM proteins with the plasma membrane (Ben-Aissa et al., 2012; Bu et al., 2020; Li et al., 2007; Orre et al., 2021) (Figs 1C and 2).

In addition to the major peptide-binding site in the F3 subdomain, an additional binding interface is present in the second β-strand in the F1 subdomain (see for example radixin in Fig. 1C). In talin, this binding interface, also present in F0, interacts with Rap1 (Fig. 2C) (Bromberger et al., 2019; Gingras et al., 2019; Zhu et al., 2017).

To prevent non-specific activation of FERM-domain-containing proteins, they form autoinhibited complexes through multiple intramolecular binding surfaces. In the case of talin, an autoinhibitory F3–R9 interface has been structurally characterized (Dedden et al., 2019; Goult et al., 2009; Song et al., 2012; Zhang et al., 2016); when mutated, it results in talin opening and integrin activation and clustering (Goksoy et al., 2008; Goult et al., 2009; Lu et al., 2022; Saltel et al., 2009; Song et al., 2012; Zhang et al., 2016). However, given that the compact form of the talin FERM domain has only been recently crystalized (Zhang et al., 2020) (see Fig. 1C, PDB 6VGU), not all the FERM–bundle interfaces in talin might have been identified (Banno et al., 2012).

These examples show the diversity and structural similarities of FERM domains, making them a highly versatile multifunctional toolbox to regulate cell shape and cell adhesion.

## The talin activation process – membrane docking of the FERM domain

Talin exists as an inactive, autoinhibited dimer in the cytoplasm (Collier and Wang, 1982; Goult et al., 2013), but neither the structure, nor the activation process of talin is fully understood. Talin activation is a highly regulated process, linking its conformational changes (opening and extending) to integrin and F-actin binding, and finally cell adhesion. The inhibitory F3–R9 interaction mentioned above masks the membrane-proximal integrin- and PIP2-binding sites in F3 (Fig. 2A,B). In addition, further autoinhibitory interactions between the FERM domain and the bundle duplex comprising R1 and R2 (hereafter R1/R2 bundle duplex), as well as F2–R12 have been proposed to shield the FERM domain from interacting with the plasma membrane (Banno et al., 2012; Dedden et al., 2019).

Talin dimerization is mediated by the C-terminal dimerization helix (Gingras et al., 2008; Smith and McCann, 2007). In addition, a R8–R9 dimerization interface has been identified, located within the ‘branched’ triad comprising R7, R8 and R9 (hereafter R7/R8/R9 triad) (Fig. 1A). This dimerization site overlaps with the binding site for RIAM, DLC1 (also known as ARHGAP7), CDK1 and paxillin in R8, pointing to mutually exclusive interactions regulating the activation of the talin dimer (Chang et al., 2014; Zhang et al., 2016).

To activate talin and to bring it to the plasma membrane, multiple mechanisms converge. A key factor in talin-mediated integrin activation is GTP-bound Rap1 (Bos et al., 2003), which interacts with the Rap1-binding RA-module in RIAM (also known as APBB1IP) (Zhang et al., 2014), but Rap1 binds also directly to the talin F0 and F1 subdomains (Gingras et al., 2019; Zhu et al., 2017) (Fig. 2C). The Rap1 interaction with tyrosine-kinase-activated RIAM (Chang et al., 2019; Cho et al., 2021) leads to the exposure of the talin-binding site (TBS) in RIAM, tethering the R8 bundle to the plasma membrane. Interestingly, this mechanism of talin membrane tethering by the exposed TBS of the Rap1–RIAM complex is only required for the talin-mediated activation of β2-integrins (LFA-1), but dispensable for activation of β1-integrin (VLA-4) receptors in leukocytes, or platelet spreading via αIIbβ3 (Bromberger et al., 2021; Klapproth et al., 2015). For platelet spreading, a direct talin–Rap1 interaction is required for αIIbβ3 activation and subsequent platelet adhesion and spreading (Bromberger et al., 2018, 2019; Lagarrigue et al., 2020; Stefanini et al., 2018). Interestingly, direct interactions between the F0 and F1 subdomains of talin and Rap1 can bring the talin-head–R9 complex very close to the plasma membrane, enabling R9 dissociation and simultaneous FERM–PIP2 association (Bromberger et al., 2019; Gingras et al., 2019; Zhu et al., 2017) (Fig. 2C).

Once the R9–FERM auto-inhibitory interaction is released, the R7/R8/R9 triad forms a hotspot of molecular interactions, such as enabling the link to microtubules via KANK1– or KANK2–R7 interaction (Bouchet et al., 2016; Sun et al., 2016), paxillin binding (Lu et al., 2022; Zacharchenko et al., 2016), focal adhesion turnover by proteolytic regulation of kindlin via CDK1 binding to R8 (Chen et al., 2022; Gough et al., 2021), as well as tensional control at focal adhesions by Rho-GAP DLC1 recruitment to R8 (Haining et al., 2018).

In summary, the interactions between Rap1–RIAM and R8, and especially also those between Rap1 and F0 or F1 tether talin to the plasma membrane, whereas R9 shields the PIP2-interacting residues in the F3 domain of the talin FERM domain (Fig. 2B). The PIP2 docking of the membrane-tethered talin FERM domain is potentially initiated by PIP2-binding sites in the F2 subdomain and F1 loop, eventually leading to dissociation of R9 from F3 and full FERM association with the plasma membrane. There, the talin F3 domain can interact with integrins (Zhang et al., 2020), diffusing in a kindlin-bound state in the membrane to form substrate immobilized integrin–talin–kindlin complexes (Fischer et al., 2021; Kukkurainen et al., 2020; Orre et al., 2021).

## The critical roles of the talin FERM domain and its F1 loop in integrin activation

**Fig. 3.**
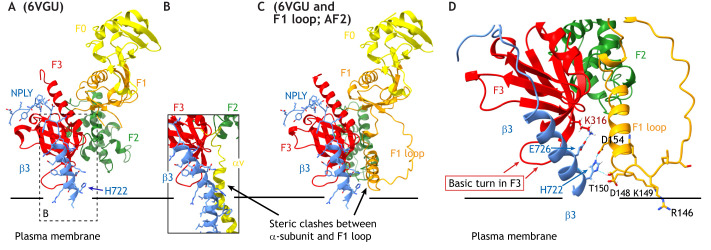
**Function of the talin head F1 loop in the context of talin-bound β-integrin.** (A) Ribbon representation of β3 integrin (blue) associated with the talin FERM domain without the F1 loop (PDB 6VGU). Model is created by merging PDB 6VGU and 3G9W to show the membrane-proximal region of the β-integrin tail. (B) Complex between the talin head and integrin heterodimer (PDB 6VGU, 2KNC). (C) Ribbon representation of the compact talin FERM domain supplemented with the AlphaFold2 (AF2) modeled F1 loop (orange) (Jumper et al., 2021; Mirdita et al., 2022). The F1 loop might sterically clash with the α-integrin subunit, indicating their competition for the β-integrin subunit. (D) Proposed interactions between the F1 loop of talin and β-integrin residues as identified by charge inversion mutagenesis (E726 and K316) (Saltel et al., 2009) and cysteine cross-linking (H722 and D154) (Kukkurainen et al., 2020).

The process of integrin activation is tightly linked to talin activation and membrane binding of the talin FERM domain. Although the isolated F2-F3 subdomains of talin have been found to be sufficient for integrin activation (Tadokoro et al., 2003) and Mn^2+^-induced integrin clustering (Cluzel et al., 2005; Saltel et al., 2009), this FERM fragment failed to induce integrin clustering in the absence of either Mn^2+^ (Kukkurainen et al., 2020) or integrin binding to fibronectin (Goult et al., 2010). Thus, although elongated structures of the talin head (Elliott et al., 2010) suggested the binding of F2-F3 to the integrin tail, this F2-F3 fragment appeared, at the same time, insufficient to mediate physiological talin-mediated integrin activation. In addition, mutations in conserved talin residues that maintain the canonical FERM fold in other FERM-domain-containing proteins, abrogated talin-induced integrin clustering (Zhang et al., 2020). Therefore, we suggest that the compact FERM domain is required for Rap1- and kindlin-dependent integrin activation and clustering (Bromberger et al., 2018, 2019; Gingras et al., 2019; Kukkurainen et al., 2020). Moreover, in the FERM fold of the talin head (PDB 6VGU; Fig. 1C), the position of the F1 loop and the Rap1-binding sites in the F0 and F1 subdomain (Fig. 2C) match their functions described above. This is less the case in the previously described elongated talin head structures. Notably, in the compact talin FERM structure, the flexible F1 loop would be positioned in a spatial manner that competes with association of the α- and β-integrin subunit (Fig. 3B,C). Therefore, it is tempting to speculate that the integrin activation defect induced by the removal of the F1 loop in talin is not only caused by a lipid-binding defect (Goult et al., 2010), but rather a failure to induce inside-out activation and dissociation of the α-subunit from the juxtamembrane β-integrin tail (see below) (Kukkurainen et al., 2020). The notion that defects owing to F1 loop deletion manifest irrespective of the Rap1–talin-FERM interaction is consistent with this hypothesis (Gingras et al., 2019; Kukkurainen et al., 2020).

There is currently no structure available that shows the F1 loop embedded within the FERM-folded talin head domain. When analyzed by nuclear magnetic resonance (NMR) in the context of the isolated F1 subdomain, the loop is entirely flexible, with a tendency to form a membrane-interacting helix (Goult et al., 2010). However, in kindlin, where the F1 loop is positioned similarly to talin, a few stable interactions with the F2 and F3 subdomains can be seen (Li et al., 2017). Thus, while our structural understanding of this loop is limited, we know that it is functionally important. Notably, the activity of the talin F1 loop can be controlled by threonine phosphorylation (Goult et al., 2010; Kukkurainen et al., 2020), thereby potentially interfering with binding to negatively charged lipids. Based on the crystal structure of the FERM-folded talin head (PDB 6VGU; see Figs 1C and 3), the F1 loop is most likely positioned in close proximity to the integrin-binding site located in the F3 subdomain (Fig. 3A). This results in the localization of the F1 loop next to the talin-bound membrane-proximal helix of the β-integrin, enabling it to interact with integrin residues that otherwise are involved in the association with the integrin α-subunit (Fig. 3C). This structural model explains the cysteine cross-linking that has been observed between the F1 loop and β-integrin tail residues, and proposes a catalytic function of the F1 loop in the opening of the integrin subunits (Kukkurainen et al., 2020) (Fig. 3D). In addition, the structure shown in Fig. 3D would explain observed interference of an F1-loop-interacting antibody with integrin binding to the F3 domain (Xing et al., 2006). Consistent with this, the F1 loop is positioned next to the lysine-rich turn in the F3 subdomain, which not only controls talin–PIP2 interaction (Saltel et al., 2009), but also the association with the membrane-proximal helix of β-integrins via charge–charge and aromatic interactions (Anthis et al., 2009; Saltel et al., 2009) (Fig. 3D).

This lysine-rich turn in the talin F3 domain is relevant for integrin activation. In comparison, the integrin-binding FERM domain of myosin-X lacks such a lysine-rich turn in F3, and thus can position integrins at filopodia, but fails to activate them, probably due to missing lipid and membrane-proximal integrin interactions (Miihkinen et al., 2021).

## The structural versatility of the compact talin head and implications for binding to its integrin ligand

**Fig. 4.**
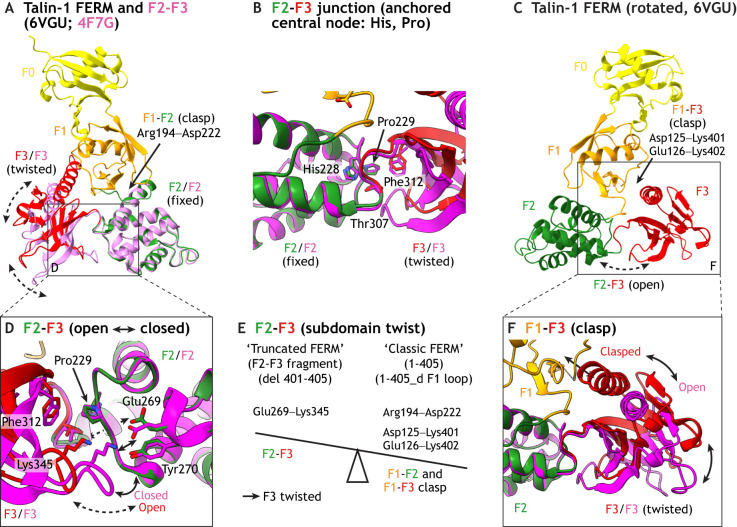
**The talin head shows structural interdomain flexibility.** (A) Ribbon model of the talin head domain in FERM conformation, represented in ‘front view’ (PDB 6VGU). The F1-F2 subdomain association (clasp) is indicated by an arrow. The F2-F3 talin head fragment (PDB 4F7G, pink) is aligned on F2, revealing a twisted orientation of F3. (B) Close-up of the ribbon model shown in A, represented by a ‘rear view’ of the center of the ‘seesaw’ in the compact FERM domain fold (PDB 6VGU; F2, green; F3, red) superimposed with a talin head F2-F3 fragment (PDB 4F7G, bright pink). Please note the overlap of the amino acids at the F2-F3 domain junction. (C) Rear view of the compact talin FERM domain showing the F1-F3 subdomain association (clasp) with an arrow, with a magnification represented in F. (D) Front view of the F2-F3 junction from A (PDB 6VGU and 4F7G), showing relevant residues and side chains in a stick representation. Please note the salt bridge (Glu269 to Lys345) that stabilizes the subdomain twist in F2-F3 talin head fragment, but not in FERM-folded talin. (E) Seesaw model describing the observed structural states of talin head and interactions specific for each conformation. (F) Zoom-in to show the F1-F3 clasp in the FERM-folded talin head (PDB 6VGU), and the twist of the F3 when this interaction is absent (PDB 4F7G, magenta).

Significant efforts have been made to determine the structure of talin and how it binds to integrins through its FERM-containing head domain (Anthis et al., 2009; Dedden et al., 2019; Elliott et al., 2010; Garcia-Alvarez et al., 2003; Goult et al., 2013; Wegener et al., 2007). The C-terminal boundary of the F3 subdomain was initially determined by proteolytic digestion, which removed the basic poly-lysine motif (residues 401–404) and was therefore excluded from the very first crystal structures of the talin F2-F3–integrin tail chimera (Garcia-Alvarez et al., 2003). The subsequent first crystal structure of the entire talin head domain, still missing this basic peptide motif 401–404, revealed an extended, linear configuration, suggesting an atypical FERM-unrelated fold of the talin head (Elliott et al., 2010). Recently, we solved the structure of a talin head containing the 401–404 poly-lysine motif that was crystallized together with the β3 integrin tail (Fig. 4A) (Zhang et al., 2020). This structure revealed a canonical FERM-like conformation and a more tightly bound β3-integrin peptide compared to the crystal structure of β3 in complex with the truncated talin F2-F3 construct (PDB 1MK7; Garcia-Alvarez et al., 2003). Interestingly, the basic motif at the end of the α1 helix in the F3 subdomain is conserved among most FERM proteins (Zhang et al., 2020). Similar to what is found for other FERM proteins, such as the ERM family or kindlin (Li et al., 2017), the 401–404 poly-lysine motif in the talin head interacts with the F1 subdomain, thus stabilizing the canonical FERM configuration (Zhang et al., 2020) (Fig. 4C,F). This F1–F3 interaction (forming a clasp) probably explains why initial talin head structures (lacking the 401–404 poly-lysine motif in F3) revealed a linear arrangement of subdomains instead of the canonical, cloverleaf FERM fold. Additional conserved interactions between the F1 and F2 subdomains further stabilize the FERM configuration (Fig. 4A), and, indeed, tampering with these interactions diminishes integrin activation and integrin clustering (Zhang et al., 2020). For these reasons, and the positioning of the F1 loop and F0-F1 domain (see above), we argue it is likely that the compact canonical FERM fold of the talin head (PDB 6VGU; Fig. 1C) is the more relevant physiological structure than the elongated conformation. However, a cryo-EM structure of full-length talin showed no density for the F0-F1 domains (Dedden et al., 2019). This could indicate that the talin head can switch between a compact, FERM fold and a linear, extended conformation (as proposed in Wen et al., 2022). Personally, we believe that the absence of a linear conformation in any other FERM-domain-containing protein and the conserved interactions stabilizing the compact talin FERM fold described above, make the extended conformation less likely. However, more experiments will be needed to examine the conformational flexibility of the talin head and the physiological relevance of different conformations.

Structural comparison of different FERM-domain-containing proteins indicate a certain flexibility, or ‘twisting’, of the talin F3 domain compared to the rest of the FERM fold. This flexibility is linked to a different set of subdomain interactions that twists the F3 position with respect to the presence or absence of the F1 subdomain, like in a seesaw. Analysis of talin structures containing the F2-F3 region only (PDB 1MK7, 3G9W and 4F7G; Anthis et al., 2009; Garcia-Alvarez et al., 2003; Song et al., 2012) or exhibiting a truncated C-terminus lacking residues 401–404 (PDB 1MK7, 3IVF and 6U4K) (Chinthalapudi et al., 2018; Elliott et al., 2010; Garcia-Alvarez et al., 2003), reveal a twisted F3 subdomain in comparison to the FERM-folded talin head (PDB 6VGU, Zhang et al., 2020) (Fig. 4E). In the intact talin FERM domain, the F3 subdomain tilts towards the F1 subdomain by forming salt-bridge interactions between the C-terminal poly-lysine motif (Lys401 and Lys402) and negatively charged Asp125 and Glu126 residues (between residues 401 and 125, and 402 and 126) (Fig. 4F), very similar to the situation in kindlin. In contrast, in the absence of the C-terminal basic 401–404 motif (PDB 1MK7), or the F1 subdomain (PDB 3G9W), the F3 subdomain twists towards the F2 subdomain and forms an atypical interactions from Glu269 and Tyr270 to Lys345 (Fig. 4D), not observed in other FERM domain proteins. In contrast, the center of the seesaw is similar in all talin crystal structures, formed by an immobile aromatic to proline interaction (Pro229 to Phe312), which is supported by a His228 to Thr307 interaction at the F2-F3 junction (Fig. 4B) (Zondlo, 2013). Owing to the missing F1 loop in the talin FERM structure (PDB 6VGU), it is so far not clear whether the F3 subdomain can twist even more, as for example seen in the kindlin structure (Li et al., 2017). The existence and relevance of such subdomain flexibility, for example in response to F1 loop movements, or kindlin–talin association, needs to be tested in the future.

## The compact FERM fold creates an open binding pocket for talin ligands

The C-terminal tail of integrins interact with the FERM domain proteins talin and kindlin, but also with adapter proteins exhibiting a classical PTB domain, such as Dok1, Dab-2 or tensin family proteins (McCleverty et al., 2007; Oxley et al., 2008; Yu et al., 2015). Switching from talin to Dok1 or Dab-2 is proposed to occur in response to integrin phosphorylation at the NPXY motif. But could there be other (structural) reasons making physiological FERM-domain interactions different from PTB-domain interactions?

**Fig. 5.**
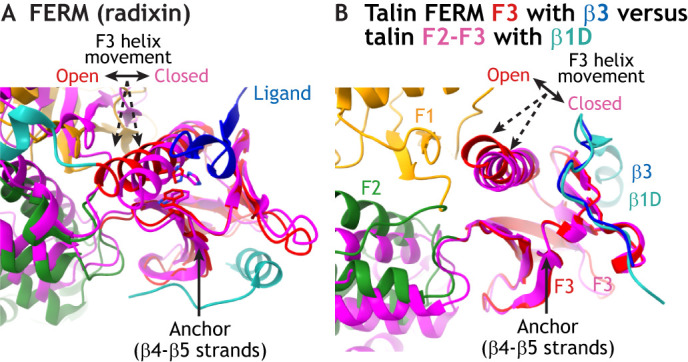
**Accommodation of the ligand-binding pocket in the FERM-folded F3 subdomains.** (A) Structural comparison of the ligand-binding pocket for F3 of closed (magenta) and open (F3, red; ligand, blue) radixin, when aligned on the β4-β5 strands of F3. Please note the lateral shift of the C-terminal α-helix in respect to the β5 strand, to accommodate ligand binding in the open conformation. (B) Structural overlay of FERM-folded talin with its bound ligand β3 integrin (PDB 6VGU; talin-1, orange-green-red; β3 integrin, blue), and the β1D-integrin-bound F2-F3 fragment of talin-2 in which the F3 helix is not mechanically coupled to the F1 subdomain (PDB 3G9W; talin-2, magenta; β1D-integrin, green-blue). Please note the ligand-directed shift in the F3 helix in this F2-F3 talin FERM fragment (double arrow), potentially linked to the particularly high ligand-binding affinity of this talin head fragment (Anthis et al., 2010).

The close association (clasp; in Fig. 4C,F) between the F1 domain and the C-terminal end of the F3 helix has consequences for the structure of the peptide-binding pocket in the PTB-folded F3 domain in FERM domain containing proteins (Fig. 5A). In closed, autoinhibited structures of FERM proteins, such as radixin, the main ligand-binding pocket in F3 is closed due to lateral movement of the C-terminal α1 helix. This induces steric clashes and prevents hydrogen (H)-bonding and full anti-parallel alignment of potential ligands with the β5 strand in F3. During FERM activation, the C-terminal α1 helix moves away from this β5 strand, opening up the F3 binding pocket for different types of peptide ligands (Figs 1C and 5A). In the case of talin, experimental data show that in the presence of the basic 401–404 motif in the C-terminal α1 helix in the F3 subdomain, the talin affinity for the β3 tail is strongly increased (Zhang et al., 2020). However, at the same time, the interaction of this basic motif with the F1 subdomain stabilizes the position of the C-terminal α-helix, keeping the NPLY-binding pocket in an ‘open’ configuration. In contrast, this C-terminal α-helix is flexible in PTB domains and in talin FERM domain fragments missing the F1–F3 association. For PTB domains, this flexibility allows the movement of the α-helix to better accommodate a bound ligand according to an ‘induced fit’ binding mode (Fig. 5B) (Zhang et al., 2020). In kindlin, the NPXY-binding pocket is also kept in a rigid and open configuration due to the F1-domain-stabilized C-terminal α-helix in F3 (Li et al., 2017). We propose that such ‘rigid-and-open’ F3 subdomain binding pockets in FERM-folded talin and kindlin are likely to be relevant for binding to integrin ligands.

On the one hand, the basic motif at the C-terminal end of the F3 helix in talin is critical for the proper folding of its NPXY ligand-binding pocket. On the other hand, the basic motif-mediated link to the F1 domain prevents the movement of the C-terminal helix towards F3-bound integrin peptides, thereby avoiding an ‘induced-fit’ conformation, seen with classical PTB-domain-containing proteins (Oxley et al., 2008). In analogy to classic FERM domain proteins, which have been shown to bind a diverse set of ligands via F3 (Fig. 1C), this rigid-and-open configuration could be designed to accommodate slightly different integrin tail sequences (β1A, β1D, β2, β3, β5, β6 or β7) via the same binding site in talin F3 (Zhang et al., 2020). This further suggests that co-recruitment of kindlin to the distal NPXY motif could strongly benefit the relatively low affinity of the rigid and open talin binding pocket for integrins. Furthermore, the open ligand-binding pocket in talin could tolerate the conformational flexibility of the integrin peptide, for example upon exposure to tension. This would allow structural variations of the talin-bound integrin peptides that could potentially be relevant for the recruitment of tension-specific integrin adapters, such as paxillin, which appears to be able to detect conformational changes of talin-bound integrin peptides (Pinon et al., 2014; Ripamonti et al., 2021; Soto-Ribeiro et al., 2019; Zhang et al., 2020).

## Mechanosignaling at the talin–integrin interface is modulated by the talin–F-actin linkage

A hallmark of integrin-dependent formation of adhesions and subsequent signaling involves the recruitment of paxillin to nascent cell–matrix adhesions at the cell periphery. Paxilin has been shown to be recruited to focal adhesions upon tension and to have a role in mechanosignaling (Kanchanawong et al., 2010; Ripamonti et al., 2021), and is thus a relevant marker to detect tensioned integrin receptors in cells (Morimatsu et al., 2015). Despite the fundamental importance of mechanotransduction, it is still not clear how the integrin–talin–kindlin complex recruits paxillin, but a two-step process has been proposed. A first step involves a kindlin-PH-domain-mediated recruitment of paxillin to nascent adhesions in the cell periphery, which is independent of talin (Theodosiou et al., 2016). Alternative paxillin–kindlin interactions at the cell edge involve the F0 subdomain of kindlin-2 binding to LIM3 and/or LIM4 of paxillin (Bottcher et al., 2017; Zhu et al., 2019). However, for full cell spreading, stress fiber formation and focal adhesion recruitment of paxillin, talin is required (Theodosiou et al., 2016). Tension-mediated paxillin recruitment to focal adhesions requires the LIM1 and LIM2 domains of paxilin, as well as the critical tyrosine residue in the talin-binding membrane-proximal NPXY motif of β-integrins (Ripamonti et al., 2021). Furthermore, when the C-terminal dimerization motif in talin is mutated, which notably affects the talin–F-actin interaction (Gingras et al., 2008), only isotropic cell spreading occurs, with a failure to form actin stress fibers and focal adhesions (Azizi et al., 2021). Likewise, a minimal talin construct, comprising only the FERM domain, the dimerization helix and F-actin-binding site in R13, induces isotropic cell spreading, without focal adhesion maturation and formation of stress fibers, thus suggesting a critical role for the second F-actin-binding domain in the talin tail for focal adhesion maturation (Atherton et al., 2015; Rahikainen et al., 2019).

Further research is required to dissect tension-dependent paxillin recruitment to talin-containing focal adhesions and to distinguish the mechanosensitive recruitment mediated by LIM domains from regulatory interactions between N-terminal paxillin LD motifs and focal adhesion proteins, such as talin, vinculin and FAK (Lu et al., 2022; Ripamonti et al., 2021, 2022; Zacharchenko et al., 2016).

## Helical bundle conversion as a mechanism to control talin–F-actin association

The talin–integrin link is found in almost all vertebrate cells and thus covers a large tensional spectrum, ranging from very soft brain tissue to highly contractile myofibroblasts. Under tension, the talin rod bundles unfold, making formerly cryptic binding sites available for vinculin recruitment in a stress-dependent manner (del Rio et al., 2009; Goult et al., 2021; Hytonen and Vogel, 2008). When comparing the tension sensitivity of the different talin rod-bundles, R8 is special, as its N-and C-termini originate and terminate in R7, providing a mechanical insulation against R8 unfolding (Haining et al., 2018). Thus, tension-mediated bundle unfolding likely follows a force hierarchy, where regulatory interactions between paxillin, DLC1 or RIAM with the R8 bundle may be the most tension resistant, as recently shown for R8–DLC1 (Dahal et al., 2022). A more thorough understanding of this emerging feedback mechanisms will be a challenging goal for future talin research.

**Fig. 6.**
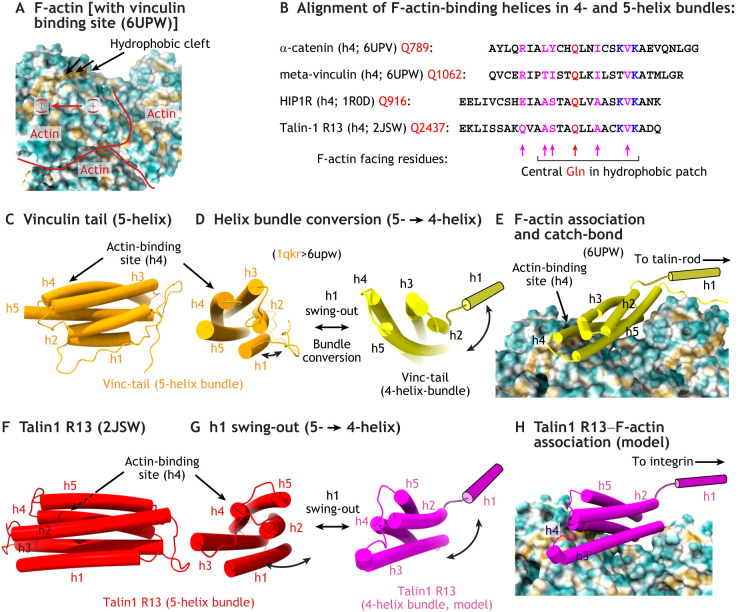
**Helix bundle conversion controls the talin–F-actin–vinculin complex.** (A) Surface structure and directionality of the F-actin polymer, visualizing the non-occupied hydrophic cleft (structure taken from PDB 6UPW, in which the bound meta-vinculin was omitted). (B) Structure-based alignment of the F-actin-binding helices shown to interact with the hydrophobic cleft, all sharing a highly conserved glutamine residue, also found within a hydrophobic patch on the THATCH domain of HIP1R and talin R13 (Brett et al., 2006). PDB codes are 6UPV, 6UPW, 1R0D and 2JSW. 4h, 4-helix bundles; 5h, 5-helix bundles. (C) Structure of the vinculin tail in the autoinhibited 5-helix state (PDB 1QKR). (D) Upon vinculin tail dissociation from the vinculin head domain, a 5- to 4-helical conversion with a simultaneous dissociation of h1 occurs, enabling the vinculin tail to fit the F-actin-binding pocket. Note potential steric clashes between h1 and F-actin in the vicinity of h5 when the vinculin tail is folded into a 5-helix bundle conformation. (E) F-actin-bound state of the 4-helix bundle of meta-vinculin as shown in A (PDB 6UPW). Note the association of the h4 helix with the hydrophobic cleft. (F) Structure of talin R13, which contains the principle F-actin-binding site in talin (Gingras et al., 2008) (PDB 2JSW). (G) Schematic representation of a possible bundle conversion of talin R13 upon h1 dissociation. (H) Structural model of talin R13 4-bundle association with F-actin, ressembling the catch-bond mechanism observed for vinculin.

Despite its critical role in the dynamic force adaptation of the talin rod bundles, the direct interaction between F-actin and talin is not that well understood. Recent studies suggest that the talin–F-actin interaction behaves like a catch-bond with a distinct directional preference (Owen et al., 2022). F-actin association with talin requires both its C-terminal 5-helix bundle (R13) and the C-terminal dimerization helix that is critical for high-affinity F-actin binding by talin (Azizi et al., 2021; Franco et al., 2006; Gingras et al., 2008; Senetar et al., 2004; Smith and McCann, 2007). However, the mechanistic details of talin–actin binding and the structural implementation of the catch-bond are not fully understood. Interestingly, Hip1R and talin R13 share a conserved glutamine residue in the F-actin binding helical bundle (Gln916 in HIPIR, Gln2437 in R13 of talin-1) (Brett et al., 2006), which is very similar to the glutamine found in the F-actin interaction site in the α-catenin and meta-vinculin (a vinculin isoform) tail domains (Mei et al., 2020) (Fig. 6A,B). In all these F-actin-binding 5-helix bundles, the h1 helix can dissociate to allow more efficient F-actin binding by the remaining 4-helix bundle due to bundle remodeling, as seen in vinculin (Fig. 6C–E). Remarkably, deletion of the h1 helix from the 5-helix R13 bundle of talin strongly increases F-actin association (Gingras et al., 2008; Senetar et al., 2004). This suggests that tension-induced interaction of vinculin and talin with F-actin is linked to the detachment of the h1 helix, leading to an enhanced association of the remaining 4-helix bundle with the retrogradely moving F-actin filament (Fig. 6F–H) (Mei et al., 2020). Detachment of h1 under tension creates an antiparallel force vector, preventing the re-association of h1 with the remaining 4-helix bundle of R13, thereby generating a catch-bond by conserving the high-affinity, F-actin-binding 4-helix bundle (Owen et al., 2022) (Fig. 6E,H). Thus, the reversible switching from a 5- to 4-helix bundle architecture is coupled to different F-actin affinities. This concept of structural bundle conversion appears to be the overarching theme of the vinculin–talin–F-actin association (Fig. 6D). In addition to tension-mediated h1 release, PIP2 interference has also been proposed to switch the vinculin tail into a 4-helix bundle (Bakolitsa et al., 1999). Therefore, both talin R13 and the vinculin tail bind to F-actin via their h4 interface, operating a catch-bond mechanism, which involves h1 dissociation and 5- to 4-helix bundle conversion.

In contrast to the R13 bundle, the second F-actin-binding site in talin R4 is not accessible under low tension. Apparently, this F-actin-binding site is exposed upon deletion of R2 and R3, mechanical destabilization of R3 or tension-mediated vinculin binding to exposed R3 helices, which all result in an enlargement of focal adhesions (Atherton et al., 2015; Rahikainen et al., 2017). Thus, tensional forces between the integrin-anchored FERM domain and F-actin-bound R13 will subsequently expose the R4 bundle to enable additional F-actin association of the talin tail domain and simultaneous exposure of vinculin-binding sites in R3.

How the creation of a second, force-bearing F-actin linkage in R4 affects adapter binding of the distal segment of the talin tail needs to be further explored.

## Conclusion

Talin is a highly complex integrin adapter protein that is modulated by membrane interactions, post-translational modifications and tension-mediated mechanical responses. Given that conformational activation of talin is highly regulated, the sequence of events leading to binding of integrin receptors, association with co-adapters such as kindlin (Fischer et al., 2021; Orre et al., 2021) and its linkage to the actin cytoskeleton require a deep understanding of the individual steps. These steps involve Rap1-mediated docking of the FERM domain to the PIP2-containing plasma membrane, followed by kindlin-assisted association with the integrin receptor, in order to induce the separation of the α- and β-integrin cytoplasmic tails via F1-loop residues. At the same time, critical regulatory mechanisms control F-actin association to allow fine tuning of cell-matrix interactions in numerous different tissue contexts. By outlining here this sequence of events we hope to stimulate further research with the goal to identify new regulatory concepts and to develop pharmacological tools that would allow to modulate the integrin–talin–F-actin linkage in pathological conditions in the future.
